# Supplementary material for: The genetic architecture of appendicular lean mass characterized by association analysis in the UK Biobank study
Source: Commun Biol. 2020 Oct 23;3:608. doi: 10.1038/s42003-020-01334-0 (PMC7585446; doi:10.1038/s42003-020-01334-0)
Supplement: Supplementary file 1 — Supplementary Information [file 42003_2020_1334_MOESM1_ESM.pdf]

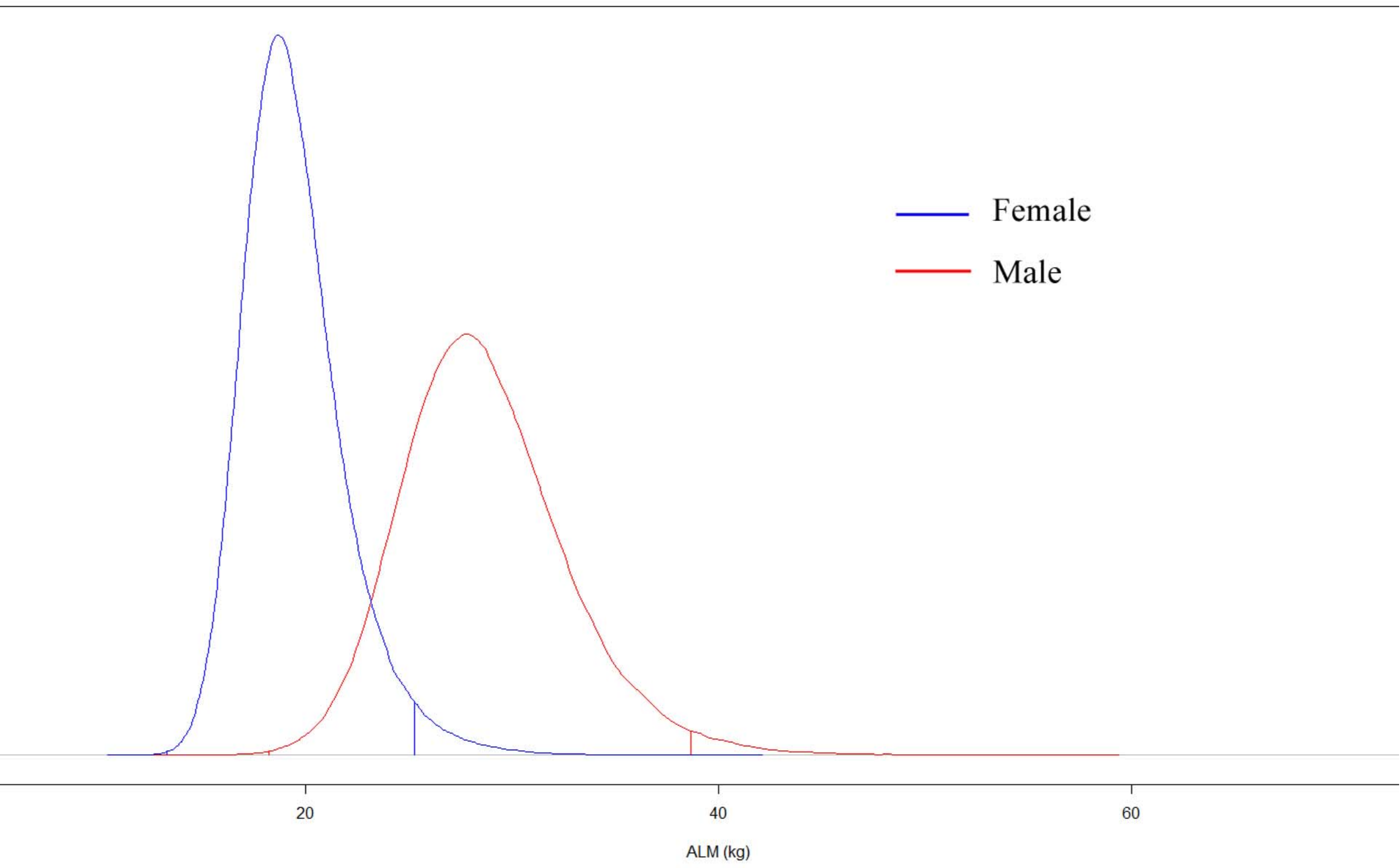
Female
Male
20
40
60
ALM (kg)

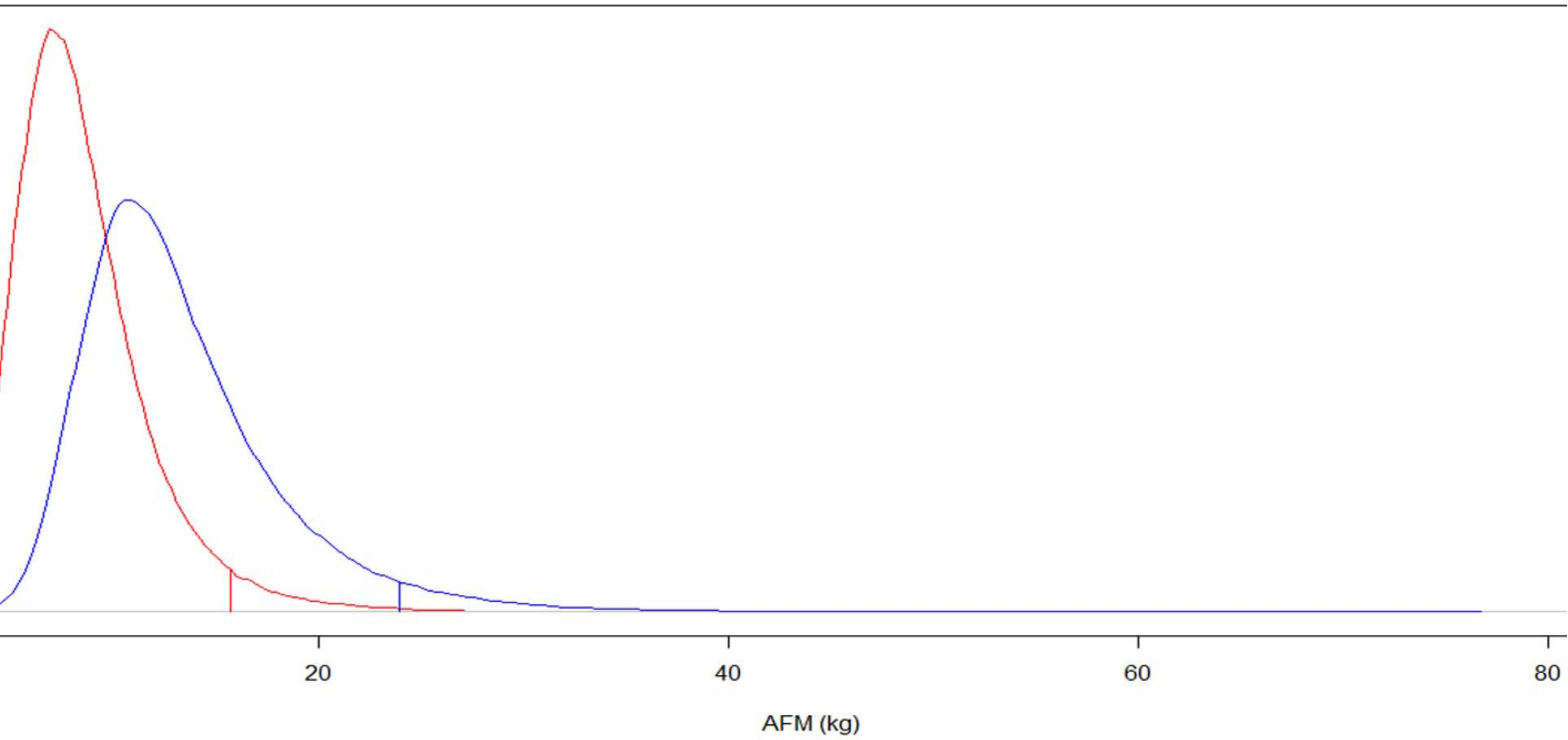
20
40
60
80
AFM (kg)

Supplementary Figure 1. Distribution of ALM and AFM

The distribution of ALM (upper) and AFM (lower) in female (blue colour) and male (red colour) participants of the UKB cohort was plotted. Vertical lines represent the phenotypic outlier thresholds defined by the Tukey (k=1.5) method.

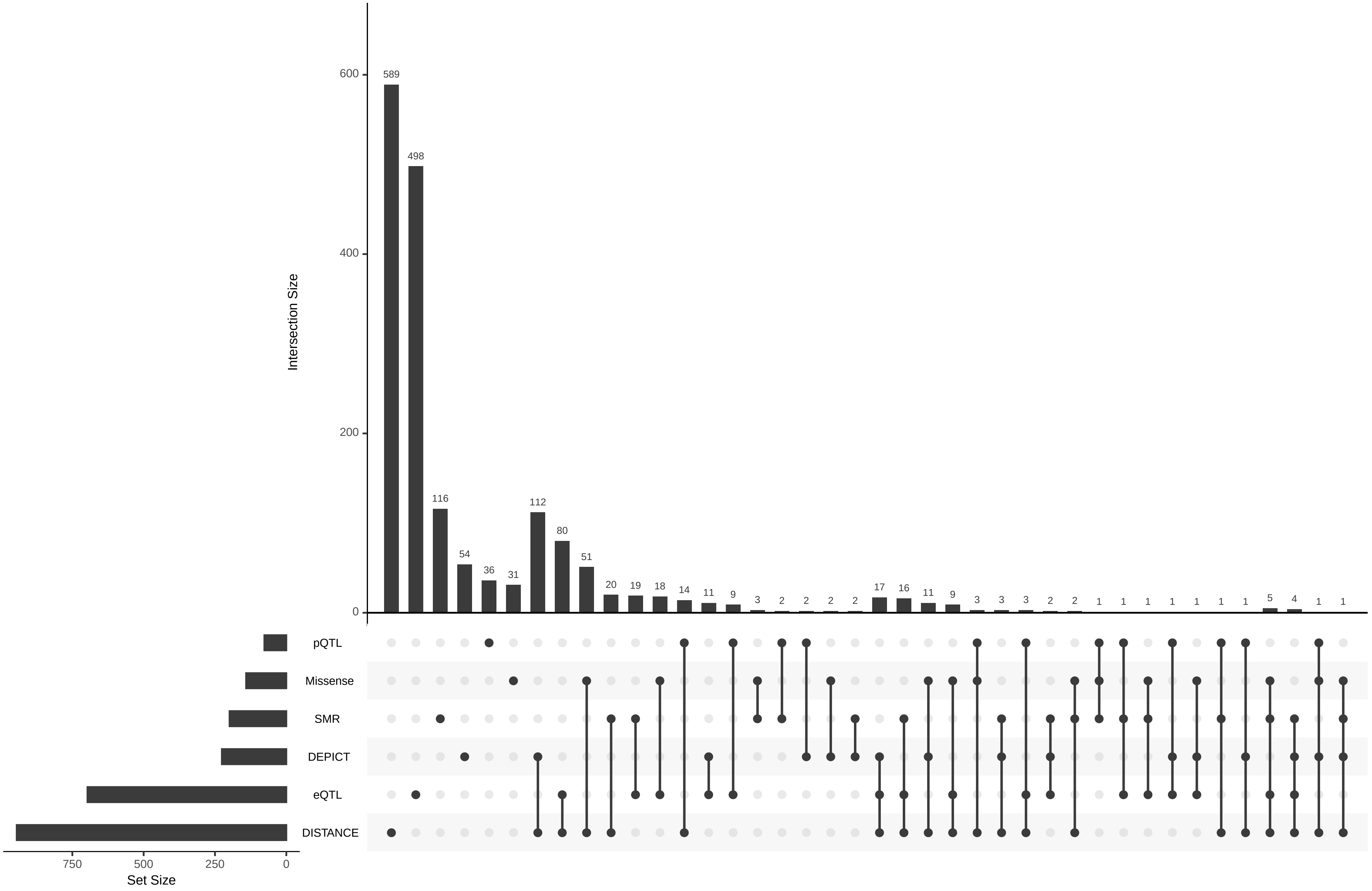
Intersection Size
600
400
200
0
589
498
116
54
36
31
112
80
51
20
19
18
14
11
9
3
2
2
2
2
17
16
11
9
3
3
3
2
2
1
1
1
1
1
1
1
5
4
1
1
pQTL
Missense
SMR
DEPICT
eQTL
DISTANCE
750
500
250
0
Set Size

Supplementary Figure 2. Intersection of genes prioritized from different sources

A total of 6 sources were used, including distance to the lead CRV, eQTL activity, pQTL activity, containing a missense CRV, DEPICT prioritization and SMR prioritization. Each row at the bottom represents one source of information. Black dots in each row indicate the presence of that source, and the co-occurrence of black dots in each vertical column indicates the intersection of the respective sources. The number of genes for each set of sources being intersected is displayed in the top panel.

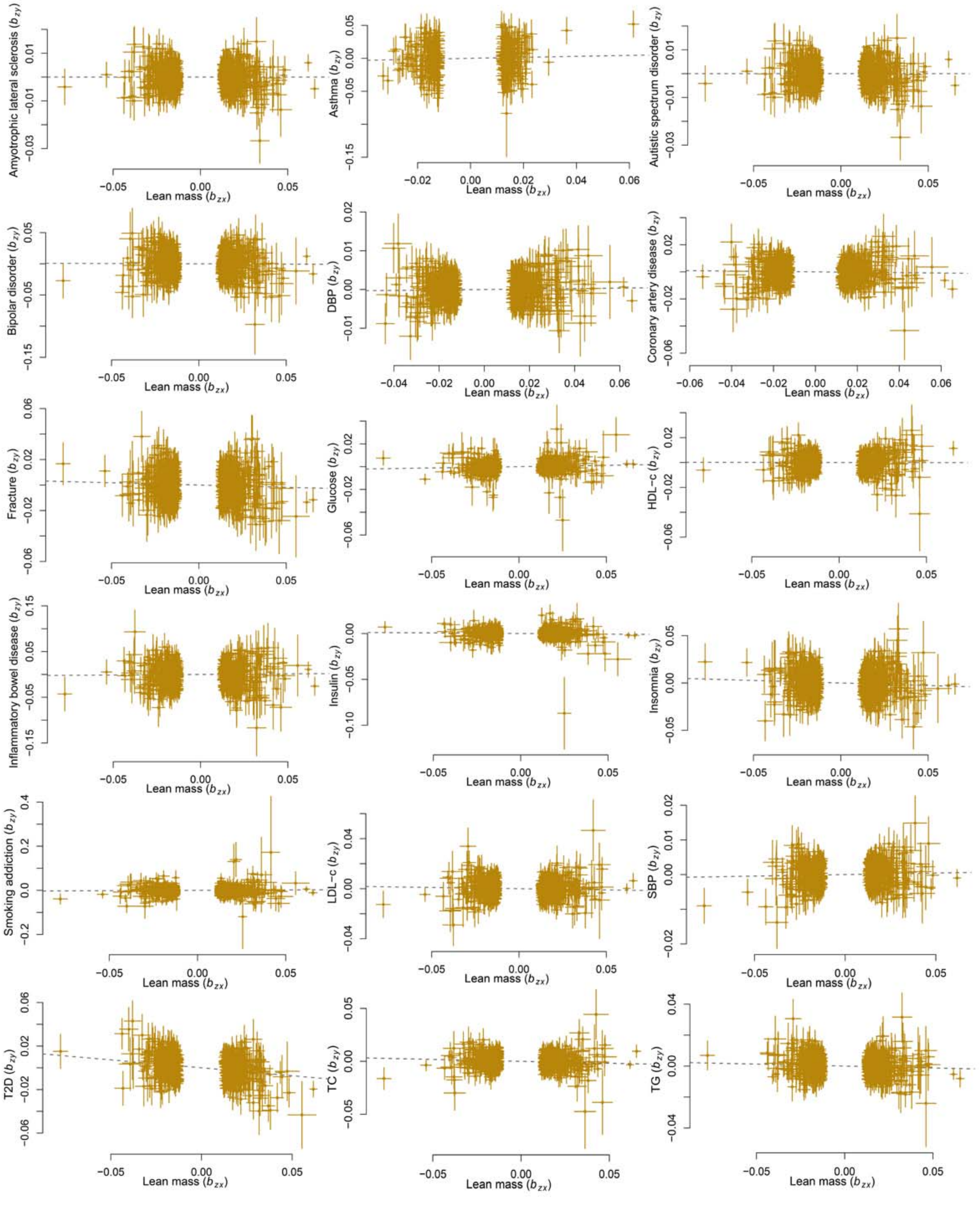

Amyotrophic lateral sclerosis (b_{zy})
Lean mass (b_{zx})
Asthma (b_{zy})
Lean mass (b_{zx})
Autistic spectrum disorder (b_{zy})
Lean mass (b_{zx})
Bipolar disorder (b_{zy})
Lean mass (b_{zx})
DBP (b_{zy})
Lean mass (b_{zx})
Coronary artery disease (b_{zy})
Lean mass (b_{zx})
Fracture (b_{zy})
Lean mass (b_{zx})
Glucose (b_{zy})
Lean mass (b_{zx})
HDL−c (b_{zy})
Lean mass (b_{zx})
Inflammatory bowel disease (b_{zy})
Lean mass (b_{zx})
Insulin (b_{zy})
Lean mass (b_{zx})
Insomnia (b_{zy})
Lean mass (b_{zx})
Smoking addiction (b_{zy})
Lean mass (b_{zx})
LDL−c (b_{zy})
Lean mass (b_{zx})
SBP (b_{zy})
Lean mass (b_{zx})
T2D (b_{zy})
Lean mass (b_{zx})
TC (b_{zy})
Lean mass (b_{zx})
TG (b_{zy})
Lean mass (b_{zx})

Supplementary Figure 3. Scatter plot of the MR analysis of the 18 traits

The x-axis represents the regression estimate of the SNP effect on ALM_{adj}. The y-axis represents the regression estimate of the SNP effect on the outcome. The horizontal and vertical lines of each cross represent 95% confidence intervals for the exposure (ALM_{adj}) and the outcome, respectively.
