# Supplementary material for: The genetic architecture of appendicular lean mass characterized by association analysis in the UK Biobank study
Source: Commun Biol. 2020 Oct 23;3:608. doi: 10.1038/s42003-020-01334-0 (PMC7585446; doi:10.1038/s42003-020-01334-0)
Supplement: Supplementary file 3 — Description of Additional Supplementary Files [file 42003_2020_1334_MOESM3_ESM.pdf]

Description of Additional Supplementary Files

File Name: Supplementary Data 1 – 17
This file contains the following Supplementary Data sheets:

Supplementary Data 1
Description: Basic characteristics of the studied UKB sample.

Supplementary Data 2
Description: Main association results in the meta-analysis of both sex groups.

Supplementary Data 3
Description: The associations of 1059 lead variants in 7,452 South Asian participants.

Supplementary Data 4
Description: The association of previously reported SNPs in the present study.

Supplementary Data 5
Description: Associations significant at the GWS level but not at the suggestive level in both sexes.

Supplementary Data 6
Description: Age-dependent effect at the 1059 lead SNPs.

Supplementary Data 7
Description: Association results of CRVs.

Supplementary Data 8
Description: Significant eQTL associations in the GTEx project data.

Supplementary Data 9
Description: Significant pQTL associations.

Supplementary Data 10
Description: Significant SMR results.

Supplementary Data 11
Description: Significant DEPICT results at FDR<5%.

Supplementary Data 12
Description: Genes prioritized by all the 6 sources.

Supplementary Data 13
Description: Comparison of imputed vs. sequenced association results on the 52 mis-sense variants.

Supplementary Data 14
Description: Deleterious prediction of the 52 mis-sense variants by 4 bioinformatical tools.

Supplementary Data 15
Description: Significant genes at the gene-based GWS level.

Supplementary Data 16
Description: Significant gene sets at the gene set based GWS level.

Supplementary Data 17
Description: Mendelian randomization analysis results by the SMR method.
