# Supplementary material for: The genetic architecture of appendicular lean mass characterized by association analysis in the UK Biobank study
Source: Commun Biol. 2020 Oct 23;3:608. doi: 10.1038/s42003-020-01334-0 (PMC7585446; doi:10.1038/s42003-020-01334-0)
Supplement: Supplementary file 5 — Reporting Summary [file 42003_2020_1334_MOESM5_ESM.pdf]

nature research

Corresponding author(s): Yu-Fang Pei, Lei Zhang

Last updated by author(s): Jun 17, 2020

Reporting Summary

Nature Research wishes to improve the reproducibility of the work that we publish. This form provides structure for consistency and transparency in reporting. For further information on Nature Research policies, see our Editorial Policies and the Editorial Policy Checklist.

Statistics

For all statistical analyses, confirm that the following items are present in the figure legend, table legend, main text, or Methods section.

n/a	Confirmed	
☐	☒	The exact sample size (*n*) for each experimental group/condition, given as a discrete number and unit of measurement
☐	☒	A statement on whether measurements were taken from distinct samples or whether the same sample was measured repeatedly
☐	☒	The statistical test(s) used AND whether they are one- or two-sided<br>*Only common tests should be described solely by name; describe more complex techniques in the Methods section.*
☐	☒	A description of all covariates tested
☐	☒	A description of any assumptions or corrections, such as tests of normality and adjustment for multiple comparisons
☐	☒	A full description of the statistical parameters including central tendency (e.g. means) or other basic estimates (e.g. regression coefficient) AND variation (e.g. standard deviation) or associated estimates of uncertainty (e.g. confidence intervals)
☐	☒	For null hypothesis testing, the test statistic (e.g. *F*, *t*, *r*) with confidence intervals, effect sizes, degrees of freedom and *P* value noted<br>*Give P values as exact values whenever suitable.*
☒	☐	For Bayesian analysis, information on the choice of priors and Markov chain Monte Carlo settings
☒	☐	For hierarchical and complex designs, identification of the appropriate level for tests and full reporting of outcomes
☐	☒	Estimates of effect sizes (e.g. Cohen's *d*, Pearson's *r*), indicating how they were calculated

Our web collection on statistics for biologists contains articles on many of the points above.

Software and code

Policy information about availability of computer code

Data collection	All data were centrally collected by the UK biobank.
Data analysis	The analysis software include: BOLT-LMM, BOLT-REML, LDSC, GCTA, PLINK, PLINK2, METAL, VEP, KING, LIFTOVER, DEPICT, SMR, MAGMA, FUMA, LDPRED, LDHUB and GSMR

For manuscripts utilizing custom algorithms or software that are central to the research but not yet described in published literature, software must be made available to editors and reviewers. We strongly encourage code deposition in a community repository (e.g. GitHub). See the Nature Research guidelines for submitting code & software for further information.

Data

Policy information about availability of data

All manuscripts must include a data availability statement. This statement should provide the following information, where applicable:

- Accession codes, unique identifiers, or web links for publicly available datasets
- A list of figures that have associated raw data
- A description of any restrictions on data availability

Genome-wide summary statistics will be made available through the NHGRI-EBI GWAS Catalog https://www.ebi.ac.uk/gwas/downloads/summary-statistics, with accession IDs GCST90000025-GCST90000027.

Field-specific reporting

Please select the one below that is the best fit for your research. If you are not sure, read the appropriate sections before making your selection.

☒ Life sciences ☐ Behavioural & social sciences ☐ Ecological, evolutionary & environmental sciences

For a reference copy of the document with all sections, see nature.com/documents/nr-reporting-summary-flat.pdf

Life sciences study design

All studies must disclose on these points even when the disclosure is negative.

Sample size	A total of 487,378 participants of the UKB cohort were used. This is the maximal number of participants who were both phenotyped and imputed.
Data exclusions	Participants who had a self-reported sex inconsistent with the genetic sex, whose sex chromosome was aneuploid, who had unusually high heterozygosity and/or missing rates or who withdrew their consents were removed. A total of 37,135 participants were. The final sample consisted of 450,243 participants, including 244,730 females and 205,513 males.
Replication	All significant findings were mutually replicated between the two sexes at the P<5e-5 level.
Randomization	NA
Blinding	NA

Reporting for specific materials, systems and methods

We require information from authors about some types of materials, experimental systems and methods used in many studies. Here, indicate whether each material, system or method listed is relevant to your study. If you are not sure if a list item applies to your research, read the appropriate section before selecting a response.

Materials & experimental systems

n/a	Involved in the study	
☒	☐	Antibodies
☒	☐	Eukaryotic cell lines
☒	☐	Palaeontology and archaeology
☒	☐	Animals and other organisms
☐	☒	Human research participants
☒	☐	Clinical data
☒	☐	Dual use research of concern

Methods

n/a	Involved in the study	
☒	☐	ChIP-seq
☒	☐	Flow cytometry
☒	☐	MRI-based neuroimaging

Human research participants

Policy information about studies involving human research participants

Population characteristics	Participants of both sexes aged between 40 and 69 were included. All participants were genotyped by the Affymetrix UK Biobank Axiom array or UK BiLEVE Axiom array.
Recruitment	UK Biobank is a prospective cohort study of over 500,000 individuals from across the United Kingdom. Participants, aged between 40 and 69, were invited to one of 22 centres across the UK between 2006 and 2010.
Ethics oversight	Ethics approval for the UKB study was obtained from the North West Centre for Research Ethics Committee (11/NW/0382), and informed consent was obtained from all participants.

Note that full information on the approval of the study protocol must also be provided in the manuscript.
